# Impact of a 20-year collaborative approach to increasing the production of rural doctors in Thailand

**DOI:** 10.5116/ijme.582f.4d3b

**Published:** 2016-12-22

**Authors:** Achara Nithiapinyasakul, Rajin Arora, Parinya Chamnan

**Affiliations:** 1 Collaborative Project to Increase Production of Rural Doctor, Ministry of Public Health, Nonthaburi, Thailand; 2 Medical Education Center, Sanpasitthirasong Hospital, Ubon Ratchathani, Thailand

**Keywords:** Collaborative approach, rural doctors, Thailand

## Introduction

Shortage and maldistribution of the health workforce has remained an important concern for health systems in many countries.^1^^-^^3^  In Thailand, different government interventions and efforts mostly through increasing production from major medical schools were successively put in place over the second half of 20th century mainly to address rural-urban uneven distribution and internal brain drain.^4^ Albeit such development, Thailand’s doctor shortage remained critical, with an approximate doctor density of 0.3 per 1,000 population in 1994^5^ and 865 medical graduates produced in that  year.^4^  Since 1994, two new government-funded projects primarily aimed to increase the production of rural doctors, called the Collaborative Project to Increase Production of Rural Doctor (CPIRD) and One District One Doctor (ODOD) program, have been employed through collaboration between the Ministry of Education and the Ministry of Public Health (MOPH). This paper describes the establishment, strategies used and the impact of the two special projects on production of doctors in rural areas.   

### Thailand’s conventional medical training

A conventional recruitment and training of medical students in Thailand, referred to as ‘a normal track’, has solely been operated by the Ministry of Education.^4^^,^^6^  Secondary school students are recruited to one of 19 medical schools based on their academic merits from the national entrance examination. Medical students under the normal track take a conventional 6-year long course of three preclinical followed by three clinical years. All students are required to pass medical school’s comprehensive examination and the national license examination to obtain their medical license. Normal track graduates are subjected to 3-year compulsory service, with 11,300 USD fine imposed for non-compliance.^6^^,^^7^ Their workplace is based on individuals’ choice provided each year’s vacancy availability.

### Establishment of CPIRD and ODOD

In order to further address the critical shortage of doctors, particularly in the remote areas of Thailand, two new government-funded initiatives called the ‘CPIRD’ and ‘ODOD’ projects were established in 1994 and 2005 respectively. They started as collaboration between the MOPH and the Ministry of Education with primary aim to develop infrastructure and strategies to increase the production of doctors who were to work for MOPH hospitals, which serves the large majority of a Thai population.^8^ A total of 14 universities are responsible for teaching pre-clinical subjects in partnership with 37 accredited MOPH hospitals nationwide teaching clinical subjects under their medical education centers.^8^

Medical students under the CPIRD are recruited from their rural domiciles, although they are mainly students from secondary schools in the provincial areas. The ODOD program extend student recruitment to those from more targeted rural and remote areas, with full government scholarship given to pursue 6-year medical training.^9^  Similar to those in the normal track, CPIRD/ODOD students must pass the comprehensive examination and the national license examination in order to obtain their medical license. Regulations on job placement, duration of mandatory service and non-adherence penalty obligation are applied. CPIRD medical graduates are subjected to 3-year mandatory service in MOPH hospitals, with substantial penalty of 11,300 USD imposed for non-adherence.  Medical graduates under the ODOD program have pre-specified job placement in their home districts and are liable for a 12-year long mandatory service in the MOPH service hospitals. Those who do not adhere to the mandatory service need to pay a substantial fine of 56,000 USD.    

### Production of medical doctors for the MOPH under the CPIRD/ ODOD projects

Up to 2015, the CPIRD/ODOD project has produced a total of 5,926 doctors for the country. The number of medical doctors under the CPIRD/ ODOD project increased from 8 in 2000 to 902 graduates in 2014. With fairly unchanged production of doctors under the normal track, CPIRD/ ODOD doctors accounted for 1% and up to 47% of a total number of newly graduates employed to work in the MOPH in employment year 2001 and 2015 respectively. Overall, 95.6% and 99.6% of medical graduates under the CPIRD/ ODOD projects passed the comprehensive examination and the national license examination, comparable to their normal track counterparts.

According to the 2015 MOPH health workforce database, 92% of medical graduates under the CPIRD/ODOD project remained working in the provinces to which they were primarily assigned. Additionally, the relative proportions of doctors under the CPIRD/ ODOD projects to total doctors who entered the MOPH over the last 15 years have consistently increased (Employment Years 2001-2015). Medical graduates under the two projects have contributed to a significant proportion of doctors working in rural areas, with them accounted for 39.0% of all doctors entering community hospitals each year. The percentage of CPIRD/ ODOD graduates to total doctors who were working for the MOPH hospitals in 2015 was 23% and varied from 12% to 38% across 12 health regions of Thailand.

The favourable effects of the CPIRD/ ODOD projects reported in this paper might be explained by a number of key strategies and policy interventions implemented. These include various educational strategies such as targeted recruitment policy to enrol students with rural background and locating medical training schools and facilities outside the capital and major cities, which have been suggested to increase the likelihood of medical graduates to choose to work in rural areas.^10^^-^^12^ Additionally, compulsory service requirements in rural and remote areas are regulated to help increase recruitment and subsequent retention of doctors in the MOPH hospitals.

Recruitment of medical students with rural background, early exposure to rural health care services and locating medical schools and training services outside major cities have been reported to help increase rural primary care practice and retention in some countries.^10^^-^^14^  In Thailand, these strategies have so far contributed to a significant increase in the number of doctors in rural areas. Further, compared to normal track graduates, CPIRD/ODOD graduates appeared to have better clinical competencies and were twice as likely to fulfil their 3-year mandatory service^6^ and continue to work in the rural areas for a longer period after mandatory service.^15^  Altogether, it is suggested that this collaborative approach through effective partnership between the Ministry of Education and the MOPH hospitals represent an effective and efficient approach to increasing the production of rural doctors and merits continuation, expansion or replication.    

 Although medical teaching under the CPIRD/ ODOD is largely carried out in service hospitals where instructors are usually overwhelmed by healthcare workload, rates of student graduation and passing the national license examination were not compromised. Medical teaching in service hospitals may also help equip students with better clinical competency as compared to teaching in large university hospitals.^6^  This is in line with the WHO Initiative on Transforming and Scaling up Health Professionals' Education and Training^16^ and the Global Commission on Education of Health Professionals for the 21st Century^17^  which emphasize that doctors should be produced in real health service system with enhancing community-oriented competencies such as teamwork skills and being a change agent.

## Conclusions

A collaborative approach to increasing the production of doctors for remote and rural areas in the CPIRD/ ODOD projects is feasible and likely efficient. Favourable effect on the country’s doctor shortage and possibly maldistribution was accomplished through various strategies, ranging from special recruitment and utilizing existing health service outside major cities as medical schools and training facilities, to early rural service exposure, followed by regulated rural placement and mandatory service.

### Acknowledgements

We thank Mr Nawapat Umthong for research data management.

### Conflicts of Interest

The authors declare that they have no conflict of interest.
